# Monitoring gestational weight gain: setting up a regional surveillance system in Italy

**DOI:** 10.1186/s12889-023-15028-9

**Published:** 2023-01-19

**Authors:** Paola Pani, Claudia Carletti, Manuela Giangreco, Alessandra Knowles, Elena Clagnan, Michele Gobbato, Stefania Del Zotto, Adriano Cattaneo, Luca Ronfani, on behalf of the Gestational Weight Survey Group (GWS group), Caterina Businelli, Maura De Grassi, Enrica Dovier, Alessandra Glavina, Valentina Lazzari, Cristina Tomasi, Giuseppa Verardi, Elisa Michelesio, Valentina Capodicasa, Alessandra Citossi, Lorenza Driul, Jessica Fasan, Chiara Mattiussi, Emanuela Vogrig, Serena Xodo, Anna Gianesini, Diletta Lorenzon, Stefania Maccor, Ilaria Pecile, Marzia Pignat, Rubina Banco, Giulia De Zuane, Silvia Raccanelli, Carmen Zampis, Fiorenza Basaldella, Giulia Boscarol, Diletta Degenhardt, Noemi Filipaz, Diandra Gaetani, Roberta Giornelli, Gloria Godeas, Rosa Valentina Zippo, Paola Cescutti, Annalisa Ianni, Caterina Stefanutti, Cristina Alloi, Francesca Magrini, Lucia Pecci

**Affiliations:** 1 Clinical Epidemiology and Public Health Research Unit, Institute for Maternal and Child Health - IRCCS “Burlo Garofolo”, Via Dell’Istria 65/1, 34137 Trieste, Italy; 2 SC Pianificazione Programmazione E Controllo Direzionale, ARCS – Azienda Regionale di Coordinamento per la Salute Regione Autonoma Friuli Venezia Giulia, Via Pozzuolo, 330 – 33100, Udine, Italy; 3 Epidemiologist, Trieste, Italy

**Keywords:** Pregnancy, BMI, Gestational weight gain, Surveillance system, Nutritional status

## Abstract

**Background:**

In many countries, including Italy, there are few national data on pre-pregnancy Body Mass Index (BMI) and gestational weight gain (GWG), despite these being important predictors of maternal and neonatal health outcomes. This dearth of information makes it difficult to develop and monitor intervention policies to reduce the burden of disease linked to inadequate BMI status and/or GWG in pregnant women. This study describes the setting up and initial implementation of a regional surveillance system on pre-pregnancy BMI and GWG.

**Methods:**

Between 1 January 2017 and 31 December 2018, anthropometric data were collected from all pregnant women accessing public health services in the Friuli Venezia Giulia region (Italy) for first ultrasound check (T1) and at delivery (T2). Anthropometric data collected at T1 (self-reported pre-pregnancy weight and measured weight and height) and T2 (measured weight and self-reported pre-pregnancy weight and height) were compared.

**Results:**

The system was able to reach 43.8% of all the women who gave birth in the region, and provided complete data for 6400 women of the 7188 who accessed the services at T1. At the beginning of pregnancy 447 (7.0%) women were underweight, 4297 (67.1%) had normal weight, 1131 (17.7%) were overweight and 525 (8.2%) had obesity. At delivery, 2306 (36.0%) women were within the appropriate weight gain range, while for 2021 (31.6%) weight gain was insufficient and for 2073 (32.4%) excessive. Only minor differences were observed between measured and self-reported anthropometric data.

**Conclusions:**

The surveillance system offers an overview of the weight status of women during pregnancy. About 1/3 of women entered pregnancy with unsatisfactory BMI and 2/3 did not achieve the recommended weight gain. This surveillance system can be an effective tool to guide public health interventions.

## Introduction

Pre-pregnancy Body Mass Index (BMI) and gestational weight gain (GWG) are important predictors of maternal and neonatal health outcomes. Both excessive BMI at the beginning of the pregnancy and excessive GWG can affect the course of gestation and the outcomes at childbirth [1–10]. Maternal obesity seems to negatively affect fetal development [11–13] as well as long term child health, increasing the offspring’s vulnerability to obesity [14, 15]. Similarly to obesity, maternal pre-pregnancy underweight is also associated with negative health effects with short and long-term consequences for the mother and the fetus [16–21].

Both excessive and insufficient GWG affect fetal growth and weight at birth [22, 23]. Excessive body mass gained during pregnancy can become persistent even in women with normal pre-gestational BMI [2, 24–27]. This condition is a major predictor of long-term obesity, and becoming pregnant again might drive the cycle towards obesity further [2, 27]. Thus, the public health burden of inadequate pre-pregnancy and pregnancy weight status is significant, extending well beyond delivery, and should be adequately addressed. The 2009 guidelines of the Institute of Medicine (IOM, now the National Academy of Medicine, NAM) provide a range for adequate weight gain for each BMI class, indicating 0.5–2 kg as normal GWG at the end of the first trimester, regardless of pre-pregnancy BMI [28].

Unfortunately, in many countries, including Italy, data on pre-pregnancy BMI and on GWG are scarce. National statistics show a constant increase of weight excess in the total adult female population (age range: 18–69) and suggest that around 22% of women in the reproductive years (18–44 years of age) [29, 30] are likely to have a BMI above the normal range (18.5–24.9 kg/m^2^) [31]. However, no specific information is available for the pregnant population subgroup. This makes it difficult to develop and monitor intervention policies aimed at reducing the burden of disease due to inadequate BMI and/or weight gain of pregnant women. The recommendations developed by IOM, addressing health workers and women of childbearing age with regards to pregnancy, include the need to implement surveillance systems to monitor weight gain during pregnancy and postpartum weight retention, collecting data on pre-conception BMI class, age and socio-economic status [28]. Particular attention, should be paid to population subgroups that are at greater risk of having high BMI, such as women with low income, low level of schooling or belonging to historically marginalized communities [32, 33]. These recommendations are also supported by the objectives identified in the latest WHO European Food and Nutrition Action Plan 2015–2020 [34].

Based on all the above, we designed and set up in one Italian region, a surveillance system that provides: 1) annual estimates and trends of the prevalence of inadequate BMI before and during pregnancy and 2) data on GWG. In addition to this, we also sought to assess the reliability of self-reported versus measured anthropometric data. This paper describes the process of setting up the surveillance system, the difficulties encountered in its implementation, and the reliability of the data collection method, also with regards to the sustainability of the system.

## Materials and methods

This surveillance system on the weight status of women in pregnancy (in a gender inclusive perspective, in this paper, the term “women” is meant as including any person capable of pregnancy), was designed and developed through a collaboration between the Regional Health Authority of the Friuli Venezia Giulia (FVG) region and the Institute for Maternal and Child Health – IRCCS ‘Burlo Garofolo’ of Trieste, a third level university and research hospital. FVG is a region in the north-east of Italy with about 1.2 million inhabitants and 8,000 deliveries per year, about 99% of which take place in public hospitals. Eleven centers, including all the public maternity units and private birth clinics of the region, plus one private antenatal clinic affiliated with the regional health system, were involved in the surveillance. Data collection began in pilot form on 1 January 2015 and the system was fully implemented by the beginning of 2016. This paper presents the results of two years of monitoring, from 1 January 2017 to 31 December 2018. Data were collected following Italian rules and regulations: women signed a standard privacy form to give consent to the routine acquisition and storage of health data. Approval to conduct the survey was granted by the FVG Regional Health Authority.

### Set up

The surveillance system was set up in collaboration with the healthcare staff of the centers involved in the project, taking into account their needs and routines. The accuracy of the reported data was assessed through periodic data extraction.

Two data collection times were established:

1. First ultrasound check (T1), between 11 and 14 weeks of gestation: women were asked to self-report their pre-pregnancy weight (PPW) and a direct measurement of weight and height was carried out by the health staff routinely in charge of providing pre-natal care. This time-point was selected because it’s when the greatest proportion of pregnant women in the Region access the public hospital system. The indications were: to measure women in light clothing and without shoes, to record weight to the nearest 0.1 kg using a SECA 877 scale, and height to the nearest 0.1 cm using a SECA 217 stadiometer. An ad hoc two-hour training was provided to the personnel involved, before starting the surveillance. Collected data were entered into the Regional hospital’s electronic clinical records system, known as G2, and extracted periodically to assess the accuracy of the measurements (i.e. number of decimals). The results of this periodic assessment were reported back to the health professionals for discussion.
2. Hospital admission for delivery (T2): women’s weight was measured before delivery using the same equipment and standardized protocol. On this occasion, women were also asked to provide self-reported pre-pregnancy weight and height for comparison with the information recorded at T1. The collected information was registered in the Certificate of Delivery Care (CeDAP), a national administrative form routinely filled in electronically by trained health care personnel after delivery, that includes sociodemographic information, data on pregnancy and delivery, and on selected maternal and neonatal outcomes. In FVG, for the specific purpose of this survey, since 2015, weight measured at delivery and self-reported pre-pregnancy weight and height are included among the information collected through the CeDAP system.

CeDAP data feed into the Regional Repository of MicroData (RRMD), an automated centralized record system that stores administrative and clinical data from the Italian National Health Service, using unique anonymous regional identification codes.

**Fig. 1:**
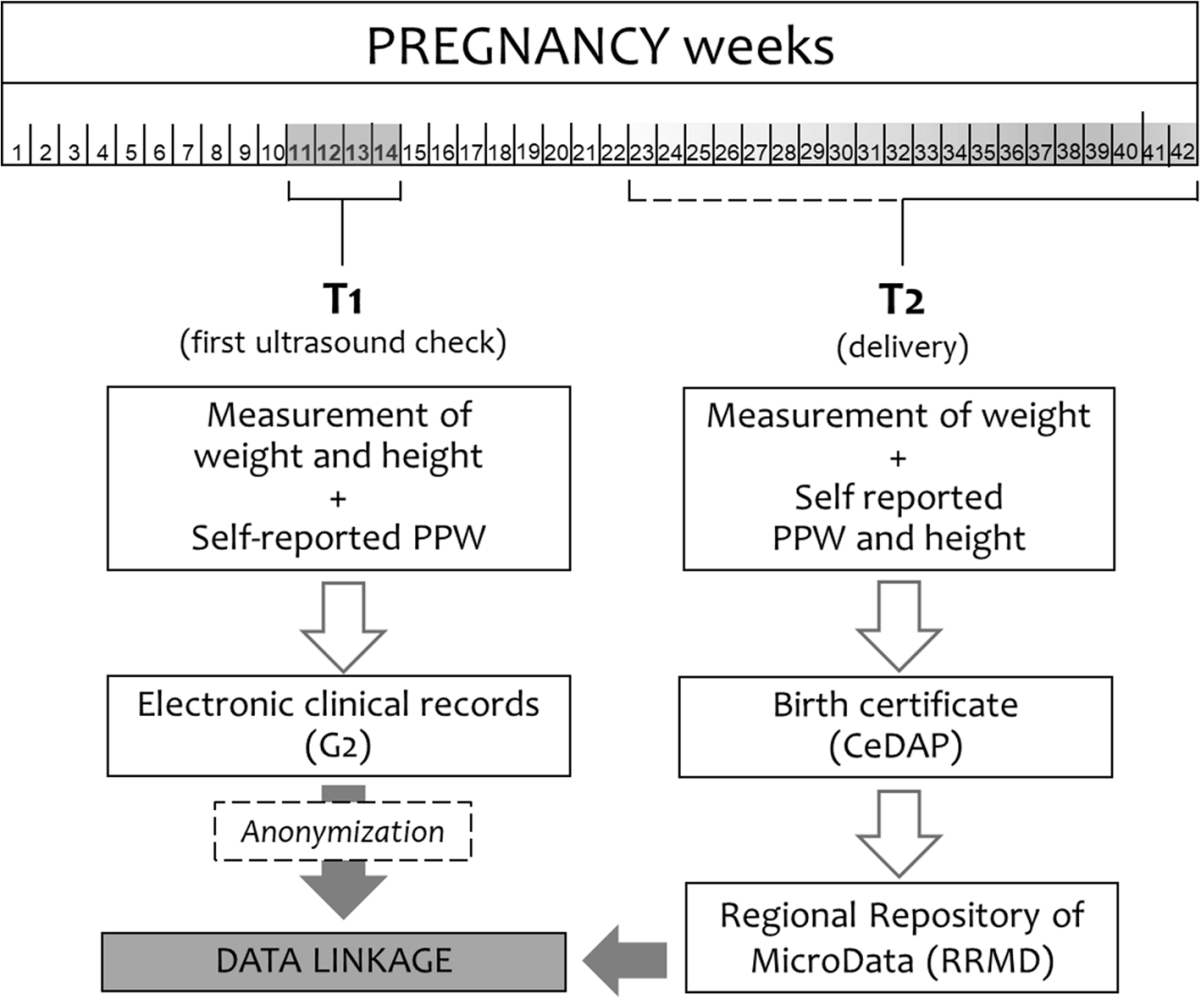
Design of the study

Anthropometric data recorded, for the purposes of this study, in the G2 system at T1, were integrated with information deriving from the CeDAP through deterministic record linkage with the databases of the Regional Repository of MicroData (RRMD) of FVG, using an anonymous identifier. A synthetic flow-chart of the study design is shown in Fig. 1.

### Population

The study population comprised all pregnant women who accessed the public health services of FVG for first ultrasound check (T1), and subsequently gave birth in the Region (T2). Only women for whom information at T1 was available, were included in the survey.

### Outcomes

The main outcome of the study was the evaluation of the weight status of the women based on pre-pregnancy BMI and GWG. Pre-pregnancy BMI was calculated as self-reported PPW in kilograms divided by measured height in meters squared, and categorized, according to WHO definitions [30], as underweight (BMI < 18.5 kg/m^2^), normal weight (BMI ≥ 18.5 kg/m^2^ to ≤ 24.9 kg/m^2^), overweight (BMI ≥ 25.0 kg/m^2^ to ≤ 29.9 kg/m^2^) and obese (BMI ≥ 30.0 kg/m^2^). The GWG was calculated by subtracting the PPW from the measured weight at delivery adjusted for gestational age, and categorized as insufficient, appropriate or excessive, based on current IOM guidelines which provide a range of adequate weight gain for each pre-pregnancy BMI class [28].

### Statistical analysis

Categorical variables are presented as absolute frequency and percentage; continuous variables as mean and standard deviation. Data on the weight status of pregnant women (pre-pregnancy BMI and GWG), based on current IOM recommendations, are presented descriptively. Differences between years were evaluated with the Chi-square test or Fisher’s exact test, when appropriate.

To evaluate the regional representativeness of the surveyed women, their socio-economic status, smoking habits during the 5 years before pregnancy, access to medical services during pregnancy, parity and pre-pregnancy BMI, were compared with those of women who had not been surveyed (women who gave birth in the Region but lacked information at T1). For continuous variables differences were evaluated with the Student t-test, for categorical variables with the Chi-square test or Fisher’s exact test, when appropriate.

To evaluate the accuracy of self-reported data and their influence on BMI classification, comparisons were carried out between: a) self-reported PPW and measured weight at T1; b) PPW self-reported at delivery and at T1; c) measured height at T1 and self-reported height at delivery; d) GWG calculated using self-reported PPW or using weight measured at T1. Differences were evaluated with the paired t-test for continuous variables, and with the weighted Cohen Kappa test for categorical variables. All analyses were performed using SAS software, Version 9.4 (SAS Institute Inc., Cary, NC, USA).

## Results

During the two-year data collection period (01.01.17 to 31.12.18), a total of 16,428 women gave birth in FVG, 7188 (43.8%) accessed the services at the first ultrasound check (T1) and were included in the assessment of the surveillance system’s accuracy and representativeness. However, only 6400 records (89.0%) had complete data and could be used to evaluate the BMI status of pregnant women. The remaining 788 records were excluded from the analysis because of missing data or because weight was ≤ 40 kg or ≥ 140 kg, and/or height was ≤ 141 cm or ≥ 198 cm.

### BMI status of pregnant women

**Fig. 2:**
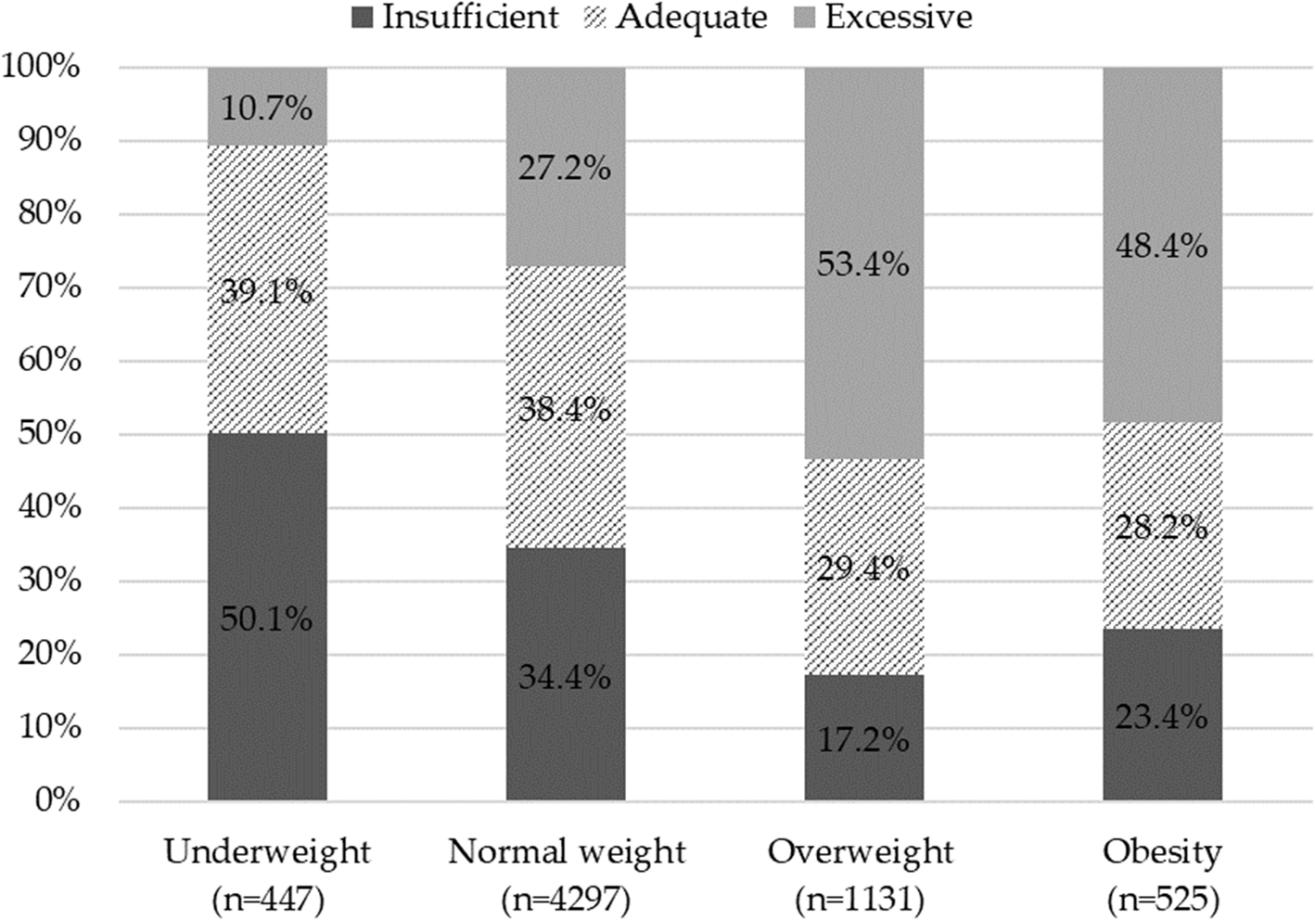
Comparison of GWG by BMI category in 2017 and 2018

At the beginning of the pregnancy, 7.0% (*n* = 447) of women were underweight, 67.1% (*n* = 4297) were normal weight, 17.7% (*n* = 1131) were overweight and 8.2% (*n* = 525) had obesity, and these percentages remained unchanged over the study period (2017 vs 2018) (*p* = 0.99). At the end of the pregnancy, 36.0% (*n* = 2306) of women had achieved adequate GWG, 31.6% (*n* = 2021) had grown insufficiently, and 32.4% (*n* = 2073) excessively. A statistically significant difference in GWG categories was observed between 2017 and 2018 (*p* = 0.04). In 2018, the percentage of women with insufficient GWG was greater than in 2017 (33.0% vs 30.2%) and the percentage of those with adequate GWG was lower (34.9% vs 37.1%), while the percentage of women with excessive GWG remained virtually unchanged (32.1% vs 32.6%). As expected, insufficient GWG was particularly marked in underweight women (Fig. 2), and this did not change between 2017 and 2018 (49.1% vs 51.2%).

Excessive GWG mostly occurred in women with a BMI in the overweight and obese range, accounting for more than 50% of the percentage distribution in both categories (Fig. 2), with a significant increase between 2017 and 2018 among women with obesity (44.9% vs 51.9%).

### Regional representativeness of women surveyed

Table 1 presents the main socio-economic characteristics of women who accessed the services at T1 (*n* = 7188), compared to those who didn’t (*n* = 9240). The differences between the two groups are more evident for the variables “Employed” and “Born in Italy”, which are higher in the survey group.

**Table 1:** Characteristics of women involved and not involved in the survey

	**Women involved** **n (%), *N* = 7188**	**Women not involved** **n (%), *N* = 9240**
Age at delivery		
< 25 years	564 (7.9)	874 (9.5)
25–34 years	4177 (58.1)	5114 (55.3)
≥ 35 years	2447 (34.0)	3252 (35.2)
Level of education		
None/completed primary school	64 (0.9)	185 (2.0)
Completed secondary school	1101 (15.3)	1473 (15.9)
Completed high school or equivalent	3381 (47.0)	4360 (47.2)
Bachelor degree or higher	2642 (36.8)	3222 (34.9)
Born in Italy		
Yes	5705 (79.4)	6477 (70.1)
Employed		
Yes	4733 (65.8)	5470 (59.2)
Parity		
Nulliparous	3705 (51.5)	4629 (50.1)
Smoking during the five years before pregnancy		
Yes	1709 (23.8)	2218 (24.0)
Medical service mainly used during pregnancy		
Public Local Family Health Unit	1151(16.0)	1578 (17.1)
Public hospital services	2605 (36.2)	3180 (34.4)
Private gynaecologist/obstetrician	2807 (39.1)	3721 (40.3)
Private Local Family Health Unit	68 (1.0)	181 (2.0)
None	3 (0.0)	22 (0.2)

### Accuracy of self-reported versus measured anthropometric data

The mean difference between self-reported PPW and measured weight at T1 was 1.45 kg (SD 2.48; *p* < 0.0001; *N* = 6393; 95% CI: 1.39–1.52) which significantly affected pre-pregnancy BMI class distribution. Despite the level of agreement being strong (Kappa = 0.82; 95% CI: 0.81–0.83), using weight measured at T1 instead of self-reported pre-pregnancy PPW to calculate the BMI, 44.8% of underweight women shifted to the normal weight category, 8.5% of normal weight women shifted to the overweight category, 5.5% of women in the overweight category downshifted to normal weight and 8.1% shifted to the obese BMI category, while 94.4% of women with obesity maintained their BMI class, as reported in Table 2.

**Table 2:**
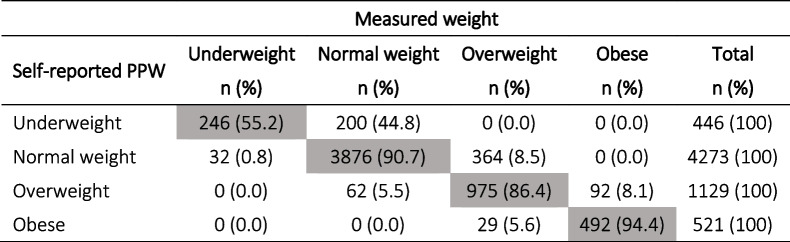
Distribution of BMI classes calculated using self-reported PPW vs weight measured at T1 and measured height at T1 for both (*N* = 6369)

Grey boxes indicate the number and % of women who remained in the same BMI class regardless of how the weight data was collected

The comparison between PPW self-reported at T1 and at delivery (T2) shows a mean difference of 0.1 kg (SD 2.10, *p* < 0.0001; *N* = 6400). As can be inferred from Table 3, this difference mostly concerned underweight women, whose pre-pregnancy BMI class shifted to normal weight in 12.2% of cases, with a strong level of agreement (Kappa = 0.91; 95% CI: 0.90–0.92).

**Table 3:**
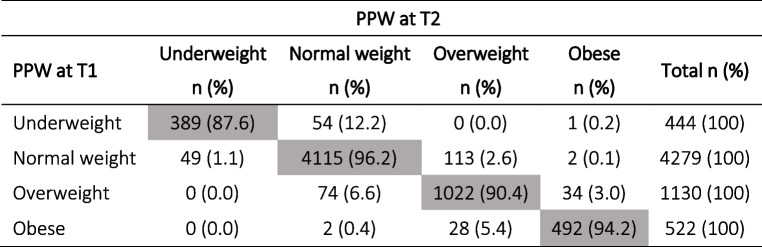
Distribution of BMI classes calculated using PPW self-reported at T1 vs PPW self-reported at T2 and measured height at T1 for both (*N* = 6375)

Grey boxes indicate the number and % of women who remained in the same BMI class regardless of when PPW data was collected

The mean difference between height self-reported at T2 and measured at T1 was 0.4 cm (SD 2.15; *p* < 0.001; *N* = 6649). Using height measured at T1 instead of self- reported height to calculate the BMI, 96.6% of normal weight women, 92.3% of overweight women and 97.2% of women with obesity maintained their BMI class, while 13.3% of underweight women shifted to normal weight (Table 4), with a very strong level of agreement (Kappa = 0.93; 95% CI: 0.92–0.94).

**Table 4:**
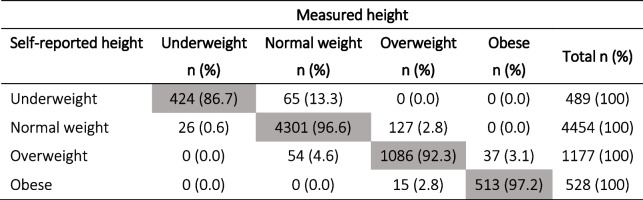
Distribution of BMI classes calculated using self-reported height vs height measured at T1 and self-reported PPW for both (*N* = 6648)

Grey boxes indicate the number and % of women who remained in the same BMI class regardless of how the height data was collected

In terms of overall GWG, using the weight measured at T1 instead of the self-reported pre-pregnancy PPW, 31.3% of women classified as having excessive GWG, shifted to adequate, 32.5% of women with adequate GWG shifted to insufficient and 10.4% of women with insufficient GWG shifted to adequate (Table 5). These differences are reflected in a weak level of agreement (Kappa = 0.53; 95% CI: 0.51–0.55).

**Table 5:**
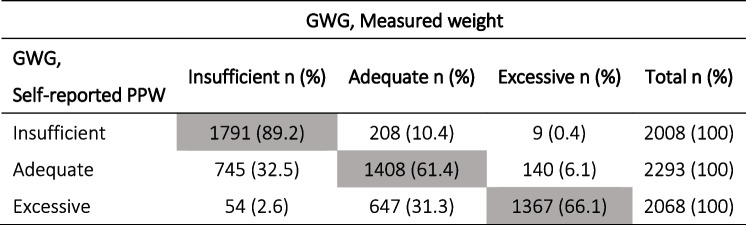
Distribution of GWG classes using self-reported PPW vs measured weights (*N* = 6369)

Grey boxes indicate the number and % of women who remained in the same GWG class regardless of how the weight data was collected

## Discussion

The results show that our surveillance system, run by health professionals routinely involved in pregnancy care and based on two different data collection times, was able to assess and monitor the anthropometric profile of pregnant women in FVG and to identify groups of women to whom integrative interventions should be targeted. To our knowledge this is the first experience in Italy of setting up a system to monitor the BMI and GWG status of pregnant women using measured data.

In FVG, 25.9% of women are overweight or with obesity at the beginning of pregnancy. Furthermore, only 36.0% of women have GWG appropriate for their pre-pregnancy BMI category, while 32.4% experience excessive weight gain. Additional relevant findings concern underweight before pregnancy and insufficient GWG, which affect 7.0% and 31.6% of women, respectively. These issues should be adequately addressed, given their possible consequences on neonatal health.

It is difficult to compare our findings with other national and international data, because this information is not available for most European countries. In the European region, the proportion of women with overweight and obesity ranges from around 30% to 50% of all women, of which between 8 and 26% have BMI ≥ 30.0 kg/m^2^ [35]. For Italy, the only available national data derive from the WHO 2009 database that includes all women of childbearing age (20 years or above) and reports an obesity rate of 15% [36]. At national level, our data can be compared to those obtained from self-reported weight and height at delivery in the CeDAP of the Emilia Romagna region. In 2017 and 2018, the overweight and obesity rate in Emilia Romagna was slightly higher than the one in FVG over the same period (27.5% vs 25.9%) [37, 38]. In Emilia Romagna, 53% of women had not achieved the recommended GWG at the end of pregnancy (22% excessive and 31% insufficient GWG), versus 64% (32.4% excessive and 31.6% insufficient) in FVG. These findings may not only be due to population differences, but also to the use of different data collection methods (self-reported only vs a mix of self-reported and actual measurements). At the international level, the overall rate of inadequate GWG observed in FVG (64%) is comparable to those described in a 2018 systematic review by Goldstein et al. for western Europe (69%), and Asia (67%), but lower than that described for the USA (72%) [39]. However, when the data are compared by GWG class, our rate of insufficient GWG is higher (32% vs 21% USA, 18% Europe, 16% Asia), while excessive GWG is lower (32% vs. 51% for all three regions).

One of the aims of our survey was to assess the accuracy of anthropometric data (both weight and height) self-reported by women at the beginning and at the end of pregnancy, and to investigate to what extent self-reported data affect the categorization of pre-pregnancy BMI. Our results show that self-reported PPW was lower than the weight measured at the first prenatal check, as would be expected from the physiological weight gain during the first trimester. In our population, the mean difference between self-reported PPW and measured weight at T1 (1.5 kg) was similar to that reported in other studies [40, 41]. The 2009 IOM recommendations indicate 0.5–2 kg as the normal weight gain for all women in the first 13 weeks of gestation [28]. Thus, our self-reported PPW at T1 appears to be congruent with both weight measured at T1 and data from the literature, thereby providing a reliable approximation of the actual PPW for the purpose of calculating pre-pregnancy BMI and GWG. Since the differences in BMI and GWG we observed are in line with the physiological GWG during the first trimester, they may not be related to PPW misreporting.

Our data also show that self-reported PPW recorded at T2 does not differ from the one collected at T1, and can thus be used as proxy for the latter. It is worth noting that also IOM recommendations on GWG [28] are based on self-reported anthropometric data, especially pre-pregnancy weight, since these values are rarely available as measured data [40, 42].

With regard to height, literature shows that it is often misreported, mostly overestimated, by women, resulting in a shift to a lower BMI class [43]. In our study, the difference between measured and self-reported height was modest (0.4 cm), affecting BMI classification only slightly, and mainly for underweight women. This finding might be explained by the fact that self-reported height at delivery could be influenced by the measurement taken at T1, which in turn can be affected by the difficulty to harmonize data collection procedures and data entering systems. Thus, while in some centers height was measured and entered correctly at T1, in others it was misregistered and rounded to the nearest integer.

A surveillance system such as the one we propose, that links anthropometric data to the socio-demographic information included in the CeDAP, can allow for the individuation of possible predictors that may have an association with maternal BMI status and GWG, as already described in other studies [33, 44]. The identification of segments of the population most at risk for under- or overweight, can usefully support policy makers and Health Authorities in planning targeted strategies of intervention.

The main limitation of our surveillance system is the unexpectedly small number of women who access public health facilities at the beginning of their pregnancy in our region, preferring private services (gynecologist/obstetrician) for antenatal care. As a result of this, the survey was able to reach only 43.8% of the population of pregnant women. The surveyed women were, however, representative of the total population of the region and their characteristics differed only modestly from those of the women who were not included in the survey: for almost all the variables considered, the differences between the two groups were of small entity.

Another limitation of this surveillance system is the long-term sustainability of the data collection effort. The healthcare staff involved in the surveillance reported experiencing difficulties with the routine acquisition and entry of the women's anthropometric data during the first ultrasound check (T1). This could explain the misregistration of measurements and missing data of the 788 women who were excluded from the analysis.

Despite its current limitations, a surveillance system such as the one implemented in FVG, is able to fulfil its purpose as an epidemiological tool to systematically monitor the weight status of women during pregnancy in the larger public context of a region, but is also flexible enough to be adapted to different settings and scales. For example, the surveillance could be simplified by collecting self-reported information on PPW at delivery, rather than during the first ultrasound check, and ensuring that height is measured during maternity stay after childbirth. This would allow the surveillance system to reach the whole of the pregnant population, regardless of whether women in pregnancy are cared for in public or private healthcare services, thereby addressing both study limitations. The system is bound to be relevant also for clinicians, who have a key role in supporting and empowering pregnant women to achieve and maintain a healthy body weight during and after pregnancy.

## Conclusions

In Italy, as in other countries, there is no systematic collection of data on maternal overweight and obesity. The only available data refer to the whole female population of childbearing age. Considering the growing obesity epidemic, and in order to pursue the objectives of the latest WHO Food and Nutrition Action Plan (2015–2020) [34], it is crucial to set up surveillance systems that can provide high quality health information to support decision-making on health practices and policies for pregnant women and new born infants.

The present experience in conducting a survey on maternal BMI during pregnancy is in line with these objectives and offers an overview of the weight status of women during the gestational period, even if only at regional level. Despite some limitations, the system allows for continuous standardized collection of anthropometric data, potentially comparable at national and international level, and can be an effective tool to guide public health interventions on maternal and child health. Further studies are needed to assess the effectiveness of our surveillance system and its reproducibility in different contexts.

## Data Availability

Restrictions apply to the availability of these data. Data were obtained from the FVG Regional Health Authority and are available from the authors with the permission of the FVG Regional Health Authority.

## Abbreviations

BMI: Body Mass Index
CeDAP: Certificato di Assistenza al Parto (Certificate of Delivery Care)
CI: Confidence Interval
FVG: Friuli Venezia Giulia
GWG: Gestational Weight Gain
IOM: Institute of Medicine of the National Academies of Washington
PPW: Pre-pregnancy weight
RRMD: Regional Repository of MicroData
SD: Standard Deviation
WHO: World Health Organization

## Declarations

### Methods

All methods were performed in accordance with the relevant Italian rules and regulations with participants signing a standard privacy form giving informed consent to the routine acquisition and storage of health data.
